# Postpartum and non-postpartum depression: a population-based matched case-control study comparing polygenic risk scores for severe mental disorders

**DOI:** 10.1038/s41398-023-02649-2

**Published:** 2023-11-13

**Authors:** Trine Munk-Olsen, Arianna Di Florio, Kathrine B. Madsen, Clara Albiñana, Merete L. Mægbæk, Veerle Bergink, Vibe G. Frøkjær, Esben Agerbo, Bjarni J. Vilhjálmsson, Thomas Werge, Merete Nordentoft, David M. Hougaard, Anders D. Børglum, Ole Mors, Preben Bo Mortensen, Xiaoqin Liu

**Affiliations:** 1 NCRR-The National Centre for Register-based Research, Aarhus University, Aarhus, Denmark; 2 iPSYCH-Lundbeck Foundation Initiative for Integrative Psychiatric Research, Aarhus, Denmark; 3 Department of Clinical Research, University of Southern Denmark, Odense, Denmark; 4 MRC Centre for Neuropsychiatric Genetics and Genomics, Cardiff University, Cardiff, UK; 5 Department of Psychiatry, Erasmus Medical Centre Rotterdam, Rotterdam, The Netherlands; 6 Department of Obstetrics, Gynecology and Reproductive Science, Icahn School of Medicine at Mount Sinai, New York City, NY USA; 7 Department of Neurology and Neurobiology Research Unit, Copenhagen University Hospital, Rigshospitalet, Denmark; 8 CIRRAU-Centre for Integrated Register-based Research, Aarhus University, Aarhus, Denmark; 9 Institute of Biological Psychiatry, Mental Health Center Sct. Hans, Mental Health Services Capital Region of Denmark, Copenhagen, Denmark; 10 Institute of Clinical Medicine, Faculty of Health Science, University of Copenhagen, Copenhagen, Denmark; 11 CORE- Copenhagen Research Centre for Mental Health, Mental Health Centre Copenhagen, Mental Health Services in the Capital Region of Denmark, Copenhagen, Denmark; 12 Department for Congenital Disorders, Statens Serum Institut, Copenhagen, Denmark; 13 Department of Biomedicine, Aarhus University, Aarhus, Denmark; 14 Center for Genomics and Personalized Medicine, CGPM, Aarhus University, Aarhus, Denmark; 15 Psychosis Research Unit, Aarhus University Hospital-Psychiatry, Risskov, Denmark

**Keywords:** Depression, Psychiatric disorders

## Abstract

It remains inconclusive whether postpartum depression (PPD) and depression with onset outside the postpartum period (MDD) are genetically distinct disorders. We aimed to investigate whether polygenic risk scores (PGSs) for major mental disorders differ between PPD cases and MDD cases in a nested case-control study of 50,057 women born from 1981 to 1997 in the iPSYCH2015 sample in Demark. We identified 333 women with first-onset postpartum depression (PPD group), who were matched with 993 women with first-onset depression diagnosed outside of postpartum (MDD group), and 999 female population controls. Data on genetics and depressive disorders were retrieved from neonatal biobanks and the Psychiatric Central Research Register. PGSs were calculated from both individual-level genetic data and meta-analysis summary statistics from the Psychiatric Genomics Consortium. Conditional logistic regression was used to calculate the odds ratio (OR), accounting for the selection-related reproductive behavior. After adjustment for covariates, higher PGSs for severe mental disorders were associated with increased ORs of both PPD and MDD. Compared with MDD cases, MDD PGS and attention-deficit/hyperactivity disorder PGS were marginally but not statistically higher for PPD cases, with the OR of PPD versus MDD being 1.12 (95% CI: 0 .97–1.29) and 1.11 (0.97–1.27) per-standard deviation increase, respectively. The ORs of PPD versus MDD did not statistically differ by PGSs of bipolar disorder, schizophrenia, or autism spectrum disorder. Our findings suggest that relying on PGS data, there was no clear evidence of distinct genetic make-up of women with depression occurring during or outside postpartum, after taking the selection-related reproductive behavior into account.

## Introduction

Women are vulnerable to mental disorders during the postpartum period, and ~10–15% of new mothers are affected by postpartum depression (PPD) [1]. PPD is not a distinct disorder in either ICD or DSM [2]. However, for DSM-5, a peripartum specifier can be applied [3]. This specifier alone covers depression onset during pregnancy and up to 4 weeks after delivery, proposing PPD as a subgroup of depressive disorders, though not considered a specific diagnostic entity. Irrespective of the lack of a distinct diagnosis, the PPD term is used daily worldwide in clinical practice and describes a group of women with depression triggered by childbirth.

As with other mental disorders, genes influence the risk of PPD and depression with onset outside the postpartum period (MDD) [4], and some studies have indicated PPD and MDD have similar genetic underpinnings, as summarized by Batt et al. [5]. However, differences between PPD and MDD have also been identified: A Swedish twin study demonstrated a heritability of perinatal depression at 44%, which was notably higher than non-perinatal depression (32%) [6]. A recent study reported a single nucleotide polymorphism (SNP) heritability of PPD of 10.7%, which was higher than the broader depression phenotype [7]. As heritability describes how much of the variation in a given trait can be attributed to genetic variation, these observations of difference in heritability could indicate PPD may be genetically distinct from depression in general. Major mental disorders, including depression, are polygenic [8], and recent advances and developments in psychiatric genetic research have applied the use of polygenic risk scores (PGS), which are measures of risk conferred by many SNPs and estimates the likelihood of a specific outcome and presenting a joint measure of genetic liability [9]. Limited evidence exists on the genetic overlap between PPD and BD, schizophrenia, and depressive episodes occurring outside the postpartum period, and the results are conflicting [7, 10–12]. While recent research has shown partially distinct PGS profiles in women with postpartum psychosis and bipolar disorder [13], only one study has directly explored and compared differences in PGS between perinatal depression and non-perinatal depression and found that perinatal depression had a higher PGS for MDD [14]. However, the cases were defined based on self-reporting and thus may be susceptible to information bias.

Based on the above-mentioned considerations, we conducted a matched case-control study to explore if PPD and MDD are distinct disorders, comparing PGSs for major mental disorders between the two groups. This was done using data on women included in the Integrative Psychiatric Research (iPSYCH) study and conducted our study through two aims: (a) to investigate whether a depression PGS can discriminate between PPD cases and MDD-matched controls; and (b) to investigate if PGSs for BD, schizophrenia (SCZ), autism spectrum disorders (ASD), and attention-deficit hyperactivity disorder (ADHD) suggest any further genetic difference between PPD and MDD, acknowledging mental disorders are polygenic, and symptoms cross diagnostic categories [15–17].

## Methods

### Data sources

**Fig. 1:**
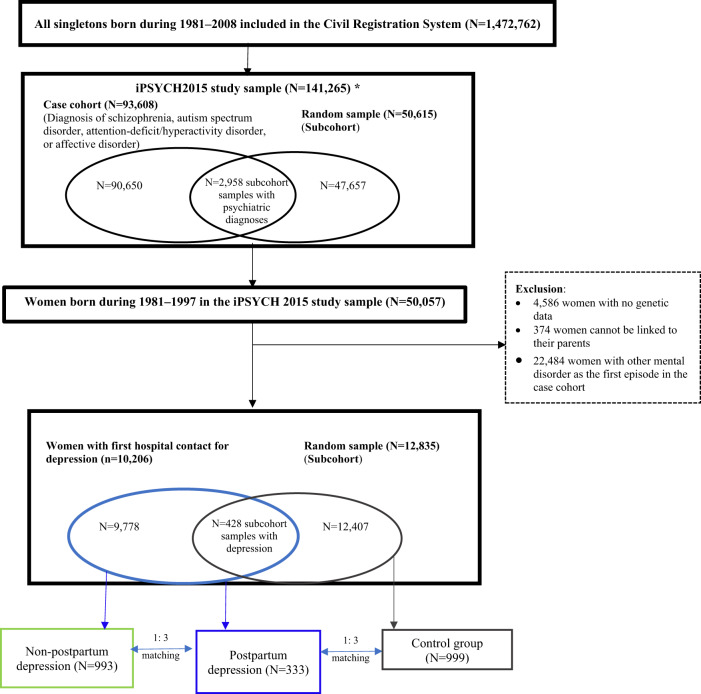
Flowchart illustrating the identification of the study population. *Ascertainment refers to whether the individuals were from the cases or the random sample of the entire Danish population.

Register data were from Danish national registers and genetic data from the iPSYCH study, based on the unique ten-digit number (CPR number) assigned to all Danish residents and registered in the Danish Civil Registration System [18]. The Danish Civil Registration System holds information on date of birth, emigration, and links to family members. The iPSYCH2015 cohort is complementary to the iPSYCH2012 cohort by expanding the study base of individuals born during 1981–2005 [19] with individuals born during 2006–2008. Therefore, the iPSYCH2015 sample is a subset of the Danish Civil Registration System of all singletons born from 1 May 1981, to 31 December 2008, who were alive and resided in Denmark at their 1-year birthday (i.e., the full cohort). Within the full cohort and through linkage to the Danish Psychiatric Central Research Register [20], all subjects with a diagnosis of SCZ, BD, affective disorders, ADHD, and ASD by the end of 2015 were identified as cases (*N* = 93,608). A random sample of 50,615 subjects (the subcohort) was drawn from the full cohort, which may also have mental disorders later (Fig. 1). A further detailed description of the iPSYCH2015 sample has been published previously [21], and in the present study, iPSYCH2012 refers exclusively to the original data selection, and iPSYCH2015i refers to the second selection, while iPSYCH2015 refers to the combined iPSYCH2012 and iPSYCH2015i. The Danish Newborn Screening Biobank stores dried blood spots taken after birth from nearly all infants born in Denmark since 1981 [22]. The Danish Psychiatric Central Research Register holds information on inpatient contacts at psychiatric treatment facilities 1969–1994 and outpatient contacts since 1995. Diagnoses were recorded using the International Classification of Diseases, 8th Revision (ICD-8) codes until 1993, and ICD-10 codes from 1994 and onwards. The Danish Medical Birth Registry was established in 1973 and holds information on the health of pregnant women and their offspring, including the date of delivery [23].

### Definition of postpartum depression and non-postpartum depression

We defined depression as a diagnosis of single and recurrent depressive disorders (ICD-8 codes: 296.09, 296.29, 298.09, and 300.49; ICD-10 codes F32–33) recorded in the Danish Psychiatric Central Research Register. The first depressive episode was defined as the date of first hospital contact for the above-mentioned diagnostic codes. Women were categorized as PPD cases if they were diagnosed with a first depressive episode within 12 months after delivery, and if contacts were outside the postpartum period, women were defined as MDD cases. We used the diagnosis of depression within 1-year postpartum to include all women with depression with postpartum onset, also women with a notable diagnostic delay between the onset of symptoms and the time of diagnosis, as recommended by the World Health Organization.

### Study population

#### PPD case identification

The iPSYCH2015 sample comprised 50,057 women born between 1981 and 1997. We excluded 4586 women with no information on genetic data or whose samples failed genetic quality control and 374 women with no linkage to their fathers. Moreover, we excluded 22,484 women with other mental disorders as the first episode from the case-cohort since these women would not contribute to the identification of PPD or MDD cases or controls. We defined other psychiatric episodes as any psychiatric diagnosis excluding major depression, substance abuse disorder, and mental retardation (290–310 in ICD-8 codes excluding 291.×9, 294.39, 296.09, 296.29, 298.09, 300.49, 303.×9, 303.20, 303.28, 303.90, and 304.×9; F00–F99 in ICD-10 codes excluding F10–F19, F32–F33, and F70–F79). Of the remaining 22,613 women, we identified PPD cases by obtaining information on the date of delivery from the Danish Medical Birth Registry (Fig. 1).

#### MDD case identification

Each PPD case was matched with up to 3 MDD cases by age at first depression diagnosis (±1 year), calendar year at birth, and year of recruitment (2012 or 2015) to account for the change of array in genotyping who were born during 1981–1997 since all PPD cases were born before 1997.

Furthermore, each PPD case was matched to 3 control women from the subcohort representing the *female background population* to consider variations in genetic liability of cases compared to a population defined as healthy controls. Criteria for matching were by year of recruitment (2012 or 2015) and calendar year at birth for women who did not have a psychiatric episode when their matched PPD case had the first depressive episode.

After matching, 333 women in the PPD group, 993 in the MDD group, and 999 women in the control group were included in the analyses.

### Polygenic risk scores

DNA was extracted from the Danish Newborn Screening Biobank, whole-genome amplified in triplicate [24]. DNA in the iPSYCH2012 sample was genotyped in 23 waves with PsychChip arrays from Illumina according to the manufacturer’s instructions and DNA in iPSYCH2015i with PsychArray V1.0 [21]. Non-genotyped markers were imputed using the 1000 Genomes Project phase 3 imputation reference panel [25]. Quality control and imputation were performed using the bioinformatics pipeline Ricopili [26].

We derived externally trained PGSs for MDD, BD, SCZ, ASD, and ADHD based on SNP weights from GWAS summary statistics excluding the iPSYCH sample [8, 27–30], using LDpred2-auto [31]. The sample size for the discovery GWASs without the iPSYCH sample used to generate the PGSs can be found elsewhere [32]. To increase the prediction ability of the PGSs, we also leveraged having individual-level SNP data on a large number of individuals with MDD, BD, SCZ, ASD, and ADHD in the iPSYCH sample by deriving another set of internally trained PGSs using BOLT-LMM [33]. These PGSs were derived using 5-fold cross-validation to avoid over-fitting of the models. The final PGSs for MDD, BD, SCZ, ASD, and ADHD were constructed as a linear combination of PGSs obtained from external GWAS summary statistics and individual-level data [32], known as meta-PGS and has been demonstrated to improve prediction accuracy for psychiatric disorders. PGS for age at first birth was calculated using LDpred2-auto with the external UK Biobank GWAS summary statistics [34] on the overlapping HapMap 3 (HM3) subset of SNPs. The number of SNPs of the different models can be seen in Stable 1 in the supplement.

### Statistical analysis

Data processing was conducted in Stata version 15.0 (StataCorp, College Station, TX, USA). Characteristics of PPD cases, MDD cases, and controls were summarized by the use of descriptive statistics.

We converted PGSs into z-scores and into quintiles according to the PGS distributions in women born during 1981–1997 from the subcohort. The standardized PGSs were calculated based on the following formula: (observed value -mean)/standard deviation. We performed conditional logistic regression to estimate the odds ratios (ORs) with 95% confidence interval (95% CI) of developing PPD or MDD versus controls by PGSs in the form of both per-standard deviation increase (continuous variable) and quintiles in comparison to the lowest quintile. The associations were compared in pairs, i.e., the PPD case with her controls and MDD cases with the controls of their matched PPD case.

The ORs of PPD versus MDD were calculated applying the following interpretation: An OR of more than 1 means the odds of being diagnosed with PPD is greater than MDD, i.e., per-standard deviation increase or a higher quintile versus the lowest quintile. All comparisons were performed only between a PPD case and the matched MDD case, therefore indirectly controlling for the applied matching criteria: age at the first depression diagnosis, calendar year at birth, and year of recruitment. We adjusted for the first 10 principal components, maternal psychiatric history, and paternal psychiatric history to account for shared familial environmental factors, and parental country of origin in the models. Maternal or paternal psychiatric history was defined as having a hospital contact for mental disorders before the PPD cases had their first episode. We performed a principal component analysis on the iPSYCH2015 sample following the guidelines in Privé et al., 2020 [35]. The Directed acyclic graph used to guide the analysis can be seen in Sfigure 1. By definition, PPD cases would have at least one child at the time of diagnosis. Any associations between PGS for mental disorders and PPD could consequently be explained by an earlier age at birth in the PPD group. To account for this, we further adjusted for PGS for age at first birth to account for the genetic correlations between mental disorders and reproductive behavior [36]. This was done based on documented differences in mental health in mothers versus non-mothers, e.g. [37] To further investigate whether any associations were driven by fertility, in the sensitivity analysis, we limited our analyses to women with at least one child at the time of first depression diagnosis and matched each PPD case to one MDD case on age at first-onset, calendar year at birth and the recruitment year (iPSYCH2012 or iPSYCH2015i) and further adjustment age at first childbirth. Altogether, 320 PPD cases were matched to 320 MDD cases. To investigate whether the associations varied by the timing of postpartum depression (PPD) diagnosis, we conducted a sensitivity analysis by categorizing PPD cases into two groups based on the onset time: early-onset (1–90 days) and late-onset (91–365 days) after delivery. Finally, as a post hoc analysis, to boost the statistical power, we meta-analyzed our results using random-effects meta-analytic models [38] to combine our results with those by Kiewa et al. [14], who investigated whether PPD and MDD are genetically distinct disorders based on 260 PPD cases and 1593 MDD cases. In their study, PPD was defined as having an Edinburgh Postnatal Depression Scale score of ≥13 within 6 months post delivery.

### Ethics

Approval for the study was obtained from the Danish Scientific Ethics Committee (Project ID: 1-10-72-287-12), the Danish Data Protection Agency (Project ID: 2012-41-0110), and the Danish Neonatal Screening Biobank Steering Committee. No informed consent is needed for register-based studies in Denmark.

## Results

The sample consisted of 333 PPD cases, 993 matched MDD cases, and 999 female controls from the background population; see Table 1 for further characteristics of the study population. All women with PPD had at least one child at diagnosis, whereas in comparison, this was observed for around 20% of women from MDD and controls at the time of matching. Women from the PPD group and MDD group were more likely to have a maternal and paternal psychiatric history than the controls from the background population.

**Table 1:** Characteristics of the study population at the time of the first psychiatric episode.

Characteristics	Postpartum depression (*n* = 333)	Major depression (*n* = 993)	Control women (*n* = 999)
**Age at first psychiatric onset (years), mean** **±** **SD**	24.9 ± 3.1	24.8 ± 3.1	-
**Parental country of origin**			
Denmark	300 (90.1)	907 (91.3)	911 (91.2)
At least one parent outside Denmark	33 (9.9)	86 (8.7)	88 (8.8)
**Maternal psychiatric history**	40 (12.0)	121 (12.2)	57 (5.7)
**Paternal psychiatric history**	37 (11.1)	106 (10.7)	51 (5.1)
**Having at least one child at the time of diagnosis**^a^	333 (100.0)	220 (22.2)	214 (21.4)
**Year of recruitment**			
2012	310 (93.1)	925 (93.2)	930 (93.1)
2015	23 (6.9)	68 (6.8)	69 (6.9)
**Calendar year at birth**			
1981–1984	174 (52.3)	521 (52.5)	522 (52.3)
1985–1989	131 (39.3)	389 (39.2)	393 (39.3)
1990–1997	28 (8.4)	83 (8.4)	84 (8.4)

Values are numbers (percentages) unless stated otherwise.

^a^For the controls, having at least one child at the time of diagnosis refers to the status when their matched PPD cases had the first episode.

### Can depression PGS discriminate between PPD and MDD (Aim 1)

**Fig. 2:**
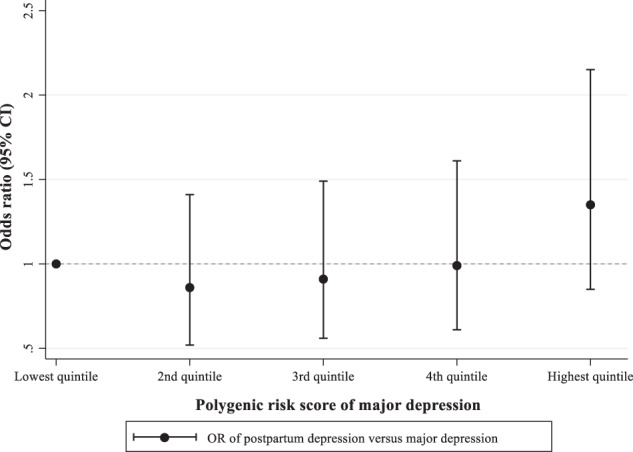
Odds ratios of PPD versus MDD by MDD PGS. OR odds ratio, 95% CI 95% confidence interval, PPD postpartum depression, MDD depression with onset outside the postpartum period, PGS polygenic risk scores. Analyses were adjusted for maternal and paternal psychiatric history, parental country of origin, polygenic risk score for age at first birth, and the first 10 principal components.

A higher MDD PGS was associated with an increased OR of PPD and MDD (STable 2 and SFigure 3). The odds of PPD versus MDD were marginally but not statistically significantly associated with a per-standard deviation increase of MDD PGS (OR = 1.12, 95% CI: 0.97–1.29). Further, the ORs did not statistically significantly differ by quintiles of MDD PGS either; the OR was for the second quintile 0.85 (95% CI: 0.52–1.39), for the third quintile 0.91 (95% CI: 0.56–1.49), for the fourth quintile 0.99 (95% CI: 0.61–1.61), and for the highest quintile 1.28 (95% CI: 0.80–2.04), in comparison to the lowest quintile (Fig. 2). The distributions of PGSs of MDD and other mental disorders and age at first childbirth are shown in SFigure 2 in the supplementary material.

### Can bipolar disorder, schizophrenia, autism, and attention-deficit hyperactivity disorder PGSs discriminate between PPD and MDD (Aim 2)

**Fig. 3:**
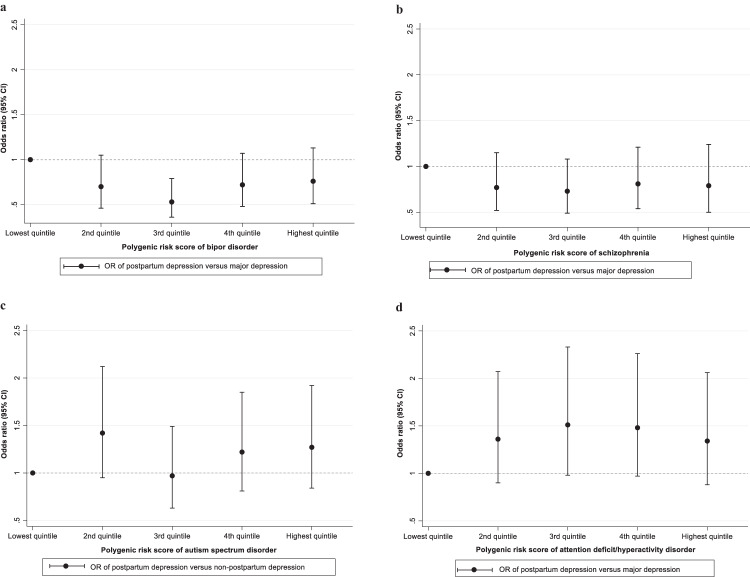
Odds ratios for association with PPD versus MDD by PGSs for bipolar disorders, schizophrenia, autism spectrum disorders, and attention-deficit hyperactivity disorder. **a** Odds ratios for association with PPD versus MDD by bipolar disorder PGS. **b** Odds ratios for association with PPD versus MDD by schizophrenia PGS. **c** Odds ratios for association with PPD versus MDD by PGS for autism spectrum disorders. **d** Odds ratios for association with PPD versus MDD by PGS for attention-deficit hyperactivity disorder. Abbreviations: OR, odds ratio; 95% CI: 95% confidence interval. PPD, postpartum depression; MDD, depression with onset outside the postpartum period; PGS, polygenic risk scores. All analyses adjusted for maternal and paternal psychiatric history, parental country of origin, polygenic risk score for age at first birth, and the first 10 principal components. ORs represent comparisons against the lowest quintile.

Both the ORs of PPD and MDD versus matched healthy controls from the background population were statistically significantly associated with the PGS for bipolar disorder but not PGS for SCZ, ASD, or ADHD (STable 2 and Sfigure 4a-4d). The ORs of PPD versus MDD were not statistically significantly associated with PGSs for our other included diagnoses: BD: 0.92 (95% CI: 0.79–1.06), SCZ: 0.89 (95% CI: 0.77–1.04), ASD: 1.05 (95% CI: 0.92–1.19), and ADHD: 1.11 (95% CI: 0.97–1.27) per-standard deviation increase (Table 2). Furthermore, when dividing the samples into quintiles, the ORs did not change by quintiles of PGSs for BD, SCZ, ASD, and ADHD (Fig. 3a–d).

**Table 2:** The odds ratios (ORs) of postpartum depression versus non-postpartum depression by per one-standard-deviation increase in the polygenic risk scores.

Polygenic risk score	Crude OR (95% CI)	Adjusted OR (95% CI)^a^
Major depression	1.16 (1.01–1.33)	1.12 (0.97–1.29)
Bipolar disorder	0.95 (0.82–1.09)	0.92 (0.79–1.06)
Schizophrenia	0.92 (0.79–1.07)	0.89 (0.77–1.04)
Autism spectrum disorder	1.03 (0.91–1.17)	1.05 (0.92–1.19)
Attention-deficit/hyperactivity disorder	1.16 (1.03–1.32)	1.11 (0.97–1.27)

*OR* odds ratio, *95% CI* 95% confidence interval.

^a^Adjusting for maternal and paternal psychiatric history, parental country of origin, polygenic risk score for age at first birth, and the first 10 principal components.

### Sensitivity analysis

When limiting our analyses to women with at least one child at the first depression diagnosis, we found that the ORs did not change by per-standard deviation increase of PGSs for MDD, BD, SCZ, ASD, and ADHD (STable 3). Our sensitivity analysis, which categorized PPD cases into two groups based on the time of onset (early-onset: 1–90 days after delivery; late-onset: 91–365 days), indicated that the associations did not differ significantly between the two groups (see STable 4). Furthermore, the meta-analysis combining our results with those from Kiewa et al.’s study [14] showed consistent findings (see Stable 5).

## Discussion

In the present study, we relied on polygenic risk scores for MDD, BD, and other mental disorders and compared women with PPD to matched female MDD controls. We found no clear evidence of genetic heterogeneity between PPD and MDD. These results jointly feed into an ongoing discussion if PPD is a distinct disorder from MDD [5, 39], and also directly into consideration if treatment for PPD and MDD should be similar, especially when new pharmacological strategies explicitly for PPD are emerging [5, 40].

We used a genetic-epidemiological perspective, exploring if genetic liability to depression and other mental disorders discriminated between our defined PPD cases and matched MDD female controls, and our results did not suggest this. One previous study reported a higher MDD PGS associated with perinatal depression than non-perinatal depression [14]. Moreover, there is extensive evidence suggesting depressive episodes with onset postpartum more often are within the bipolar spectrum than depressive episodes at other time points [41], but we did not find any difference in the PGSs for MDD or BD either, as both PPD and MD were equally associated with PGSs. There are several potential alternative explanations for the observed non-significant associations, including limited sample size. Further, the training SNPs may not be sufficient to generate PGSs to identify subtle differences between PPD and MDD, especially considering the complex genetic architectures behind mental disorders. Adding to this, we acknowledge psychiatric diagnoses do not represent natural boundaries, and PPD is an umbrella term encompassing several distinct phenotypes occurring in the postpartum period, and timing at onset may define part of the heterogeneity. Similarly, MDD is also a heterogeneous entity, and different disease courses have been reported [42]. The heterogeneity of both PPD and MDD would eventually blur the potential PGS differences between PPD and MDD. Finally, an alternative explanation is that PPD is a subgroup of MDD with the only difference that these disorders manifest in the postpartum period.

Biological studies have produced substantial evidence for distinct features and biological differences between PPD and MDD, which may, so far, represent the most compelling evidence for PPD being distinct from MDD, as reviewed by Batt and colleagues [5]. In addition, our previous study indicates that the prevalence and incidence differ before, during, and after pregnancy [39]. To explore if there is a distinct biology underlying depression with specific onset in the postpartum period could be approached through epidemiological studies, which, e.g., would study if risk factors for PPD and MDD are different or if short and long-term outcomes between the two groups are distinguishable [41, 43]. Another approach could be to compare symptomatology or study whether there is a peak in the incidence of depression after childbirth compared to other time points [39], given that the postpartum period is characterized by hormone and immune-related biological changes in the body. Finally, we also note that basic components of disease risk can be broken into genetic liability, but also environmental exposures, gene-environment interaction, and lifestyle factors, with the three latter components receiving no or little attention in several of the above-mentioned studies.

### Interpretation of our findings

Childbirth is a potential trigger of various mental disorders, including depression [1]. We join the discussion and here argue for the relevance of PPD as a specific diagnostic entity regardless of the negative findings in the present study. Defining and categorizing women with depression triggered by childbirth as a distinct disorder rather than depression offers opportunities for both research programs, clinical practice, and women themselves [2]. Recent development in specialized PPD treatment similarly builds on the rationale that women with postpartum depression require other types of care than women with depression outside the postpartum period.

### Methodological considerations

This study has several strengths, including a novel approach that relied on population data. Although our study, to our knowledge, is the first to assess the differences in PGSs between PPD and MDD, our study has limited power to detect any potential subtle differences or explore this question further by various PPD phenotypes, including early versus late-onset PPD or depression in the pregnancy. Moreover, our study population is young, with a mean age of 25 years. Consequently, most MDD cases and female population controls in the original iPSYCH dataset did not have a child, and our findings may not be generalizable to women of different age groups. However, we disentangled the effect of reproductive behavior by taking the inbuilt selection into motherhood into account by adjusting for PGS for age at first childbirth and restricted our analyses to women with at least one child at the time of first depression diagnosis, and the results remained consistent.

## Conclusion

Relying on PGS data and applying a matched study design, we did not find indications for distinct genetic contributions to PPD and MDD, even after accounting for the selection related to early age at first birth in the PPD group. Furthermore, our meta-analysis and combining our results with those from Kiewa et al. confirmed our findings. However, additional points must be raised in connection with these results: Firstly, it should be further explored how PGSs measures sufficiently capture genetic differences between PPD and MDD, and secondly, our results do not rule out other distinct features between PPD and MDD, including differences identified through epidemiology and symptomatology as well as underlying biology and the need for differential treatments.

### Supplementary information

Supplementary

## Data Availability

The data in this project were delivered by the registry holders to the researchers as pseudonymized data files. Data are available upon request to the registry holders, provided legal and ethical approvals.
