# Supplementary material for: Postpartum and non-postpartum depression: a population-based matched case-control study comparing polygenic risk scores for severe mental disorders
Source: Transl Psychiatry. 2023 Nov 13;13:346. doi: 10.1038/s41398-023-02649-2 (PMC10641081; doi:10.1038/s41398-023-02649-2)
Supplement: Supplementary file 1 — Supplementary [file 41398_2023_2649_MOESM1_ESM.docx]

**Supplementary**

**Stable 1.** Characteristics of polygenic risk scores

| **Genetic risk scores** | **Number of SNPs overlapping Hapmap3** | |
| --- | --- | --- |
|  | **External GWAS summary statistics** | **Individual-level data** |
| **Major depression** | **1,108,585** | **1,118,443** |
| **Bipolar disorder** | **1,109,886** |  |
| **Schizophrenia** | **1,097,826** |  |
| **Autism spectrum disorders** | **1,102,036** |  |
| **Attention deficit hyperactivity disorder** | **1,044,593** |  |
| **Age at first birth** | **1,063,208** | **-** |

**Sfigure 1.** Adapted directed acyclic graph used to guide the analysis


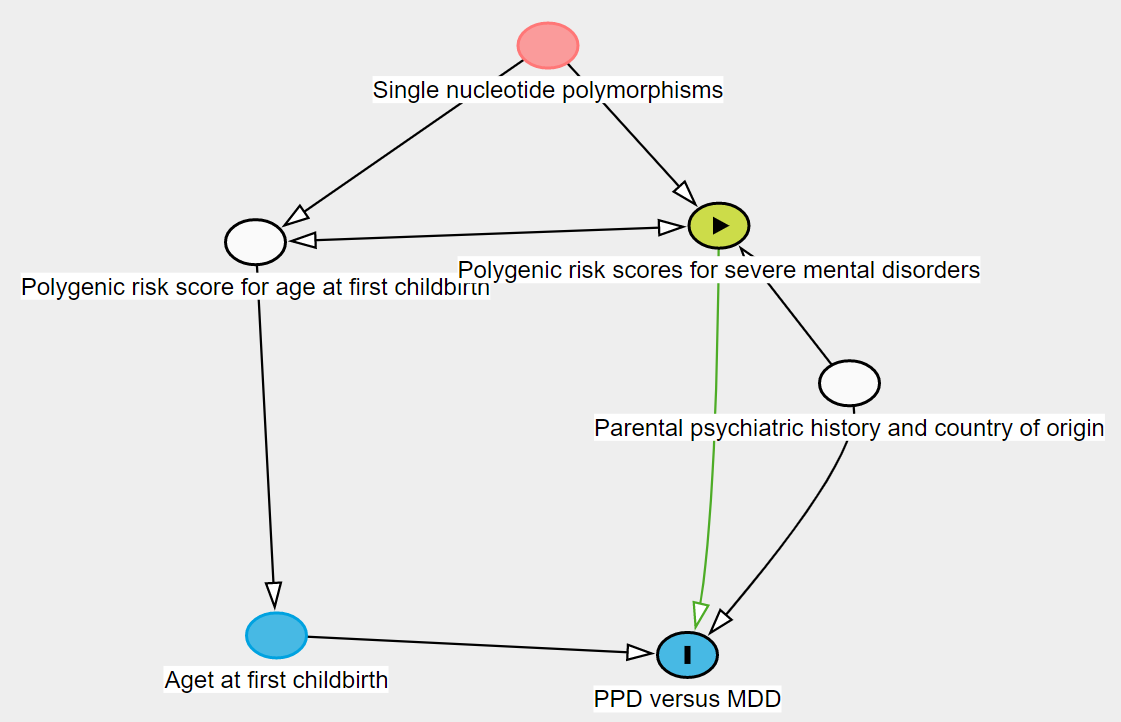


**Sfigure 2.** Kernel density distribution of PGSs in postpartum depression cases, major depression cases, and controls.

**SFig 2a**. PGS_MDD_


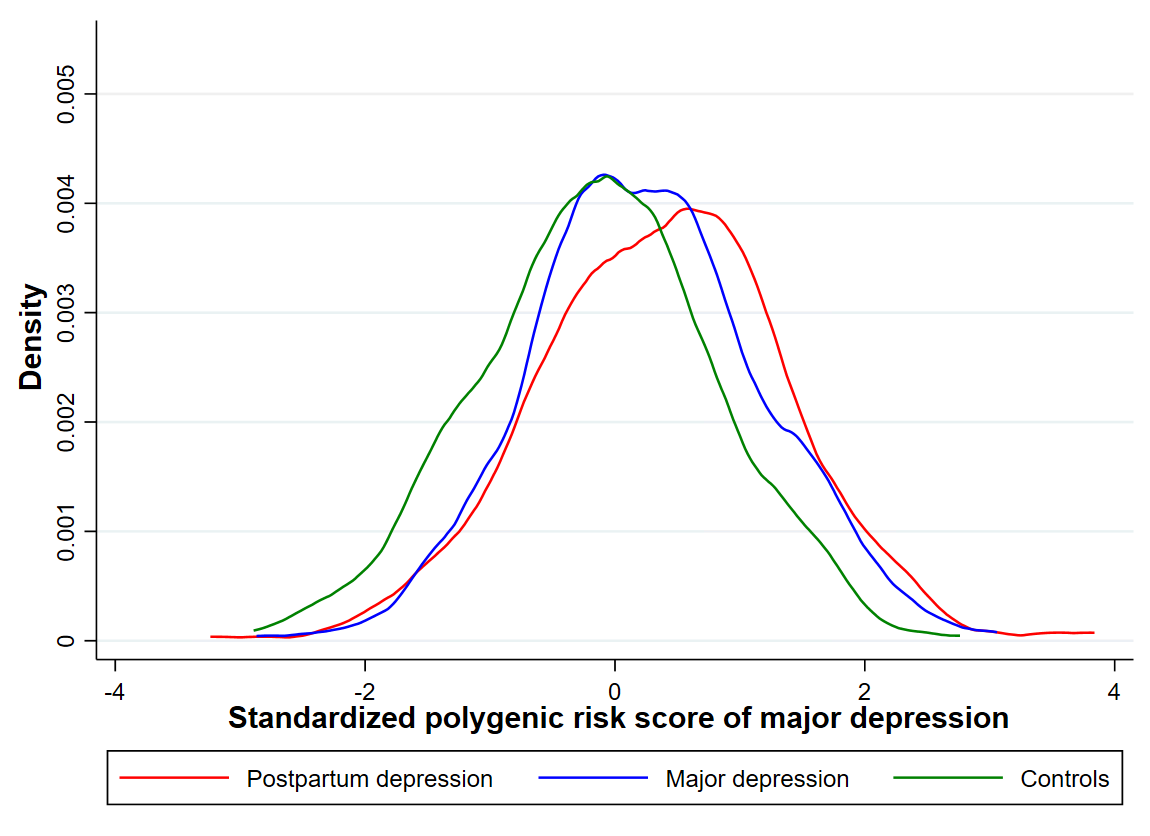


**SFig 2b**. PGS_BIP_


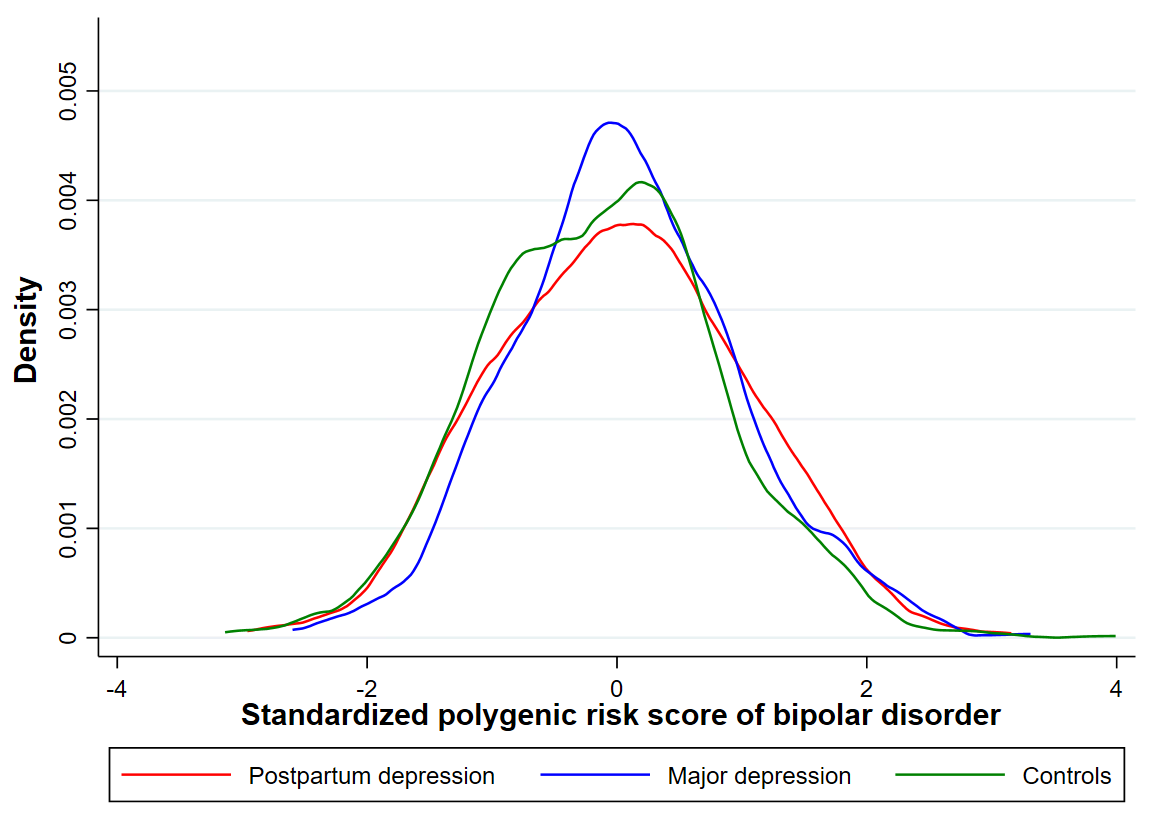


**SFig 2c**. PGS_SCZ_


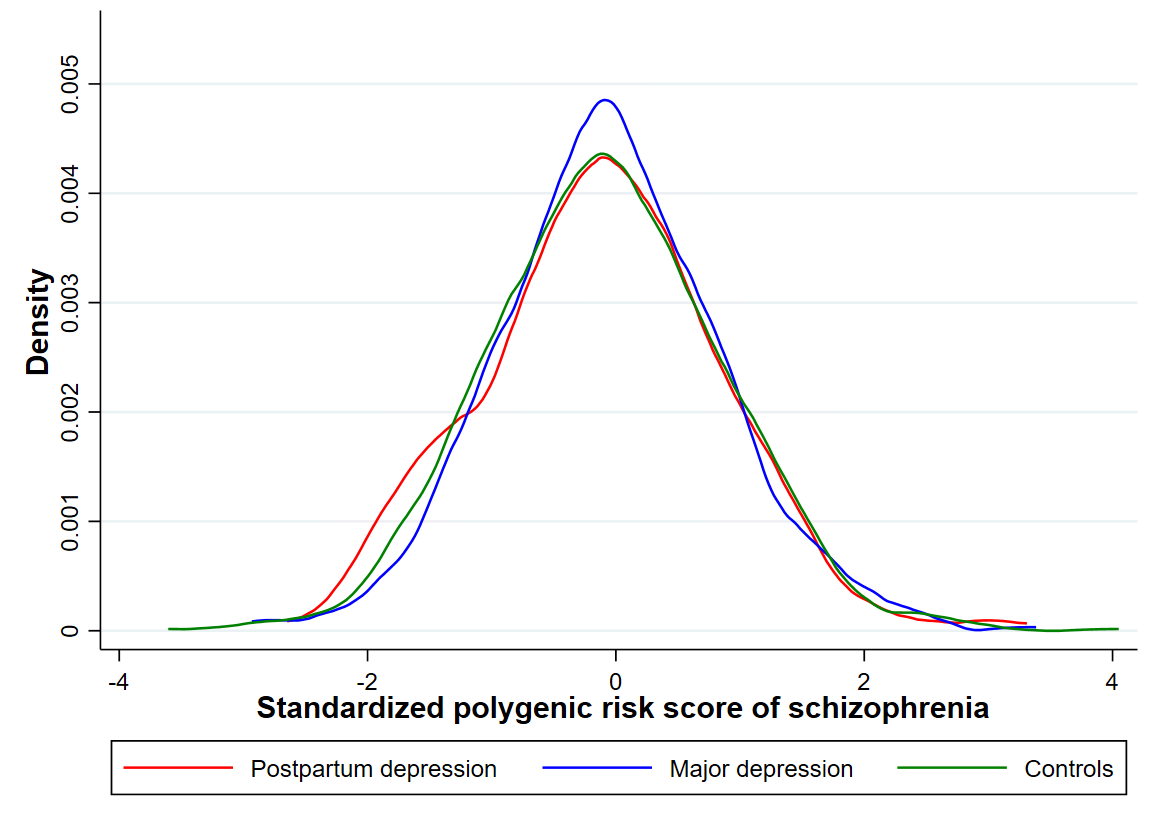


**SFigure 2d**. PGS_ADHD_


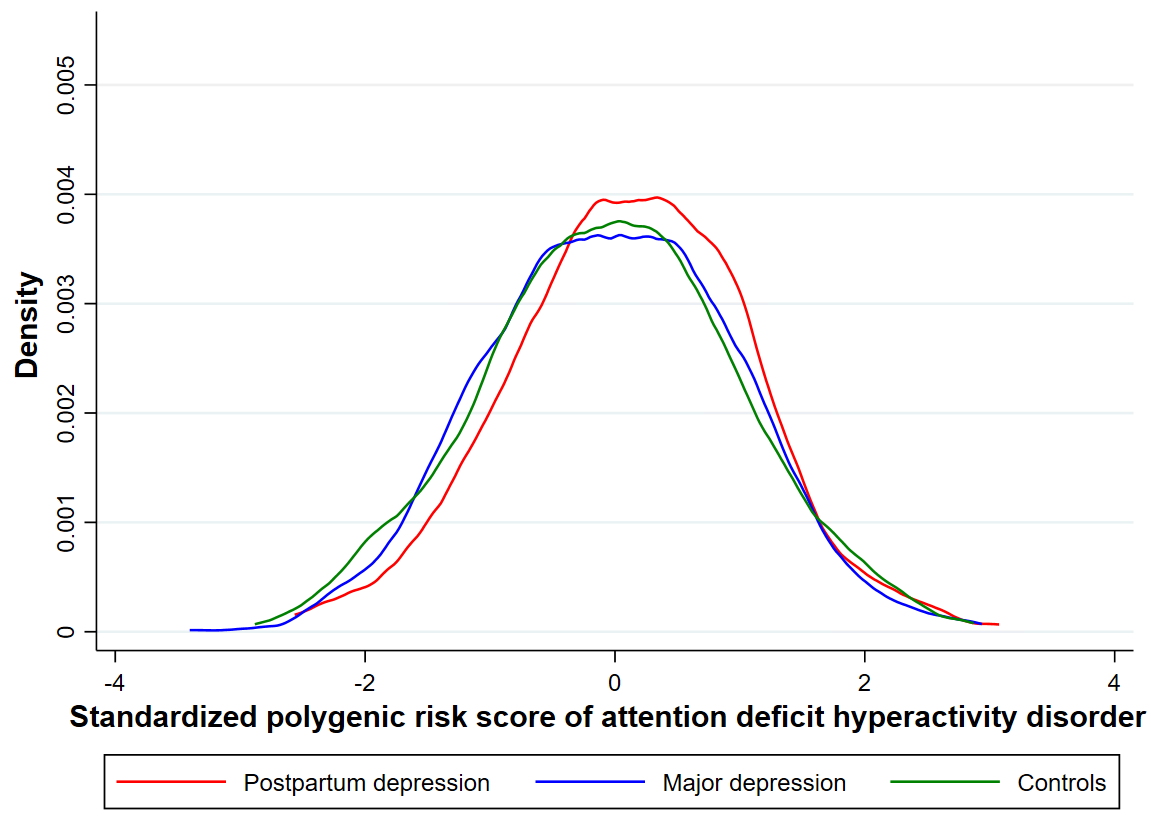


**SFig 2e**. PGS_ASD_


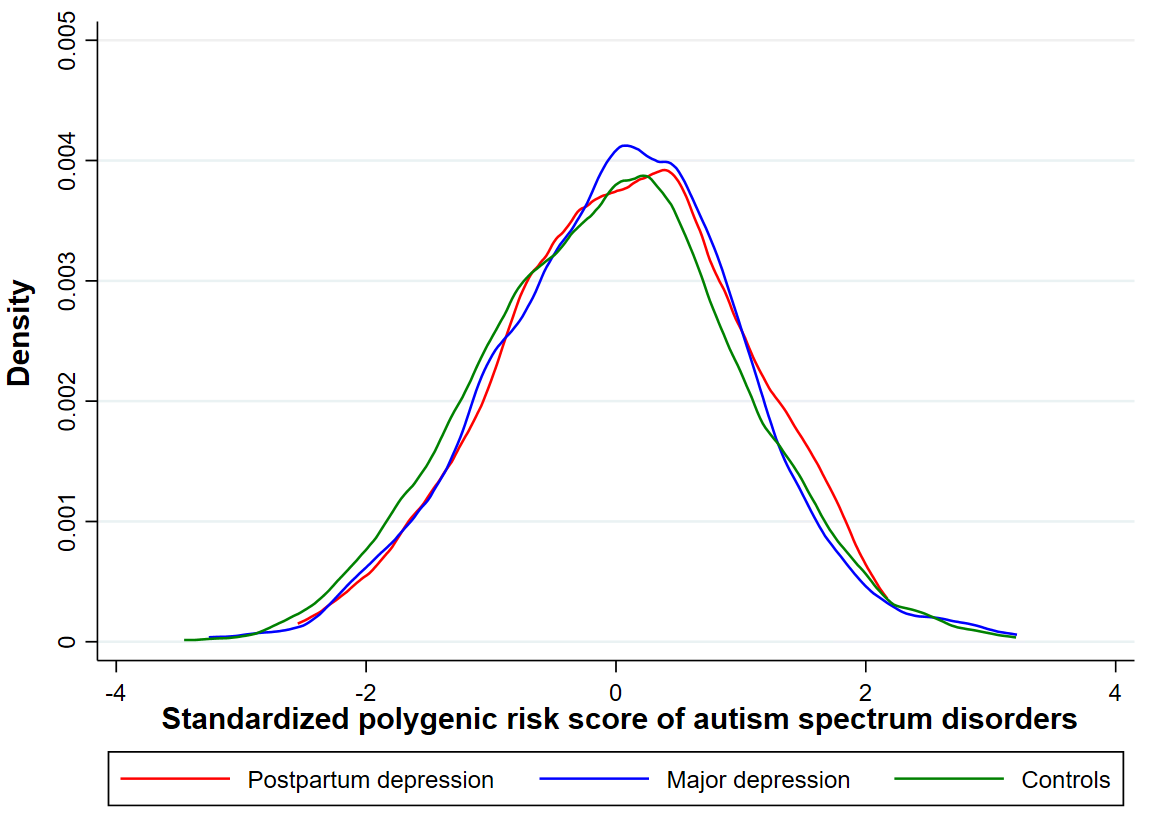


**SFig 2f**. PGS for age at first childbirth

**STable 2.** Odds ratios (ORs) of postpartum depression and major depression versus controls by per one-standard-deviation increase in the polygenic risk scores

| **Polygenic risk score** | **OR of postpartum depression vs. controls** | | **OR of major depression vs. controls** | |
| --- | --- | --- | --- | --- |
|  | **Crude OR (95% CI)** | **Adjusted OR (95% CI)** | **Crude OR (95% CI)** | **Adjusted OR (95% CI)** |
| **Major depression** | 1.79 (1.55–2.06) | 1.76 (1.51–2.05 ) | 1.58 (1.43–1.75) | 1.56 (1.41–1.72) |
| **Bipolar disorder** | 1.16 (1.02–1.32) | 1.15 (0.99–1.34) | 1.24 (1.13–1.37) | 1.28 (1.16–1.41) |
| **Schizophrenia** | 0.96 (0.84–1.10) | 0.91 (0.79–1.05) | 1.04 (0.94–1.15) | 1.03 (0.93–1.14) |
| **Autism spectrum disorder** | 1.11 (0.99–1.26) | 1.11 (0.98–1.26) | 1.08 (0.99–1.18) | 1.07 (0.98–1.17) |
| **Attention deficit/ hyperactivity disorder** | 1.17 (1.03–1.32) | 1.08 (0.95–1.23) | 1.01 (0.93–1.10) | 0.93 (0.89–1.07) |

^a^ adjusting for maternal and paternal psychiatric history, parental country of origin, polygenic risk score for age at first birth, and the first 10 principal components.

**SFigure 3.** Odds ratios (ORs) of postpartum depression and major depression versus controls binned by MDD PGS after adjusting for maternal and paternal psychiatric history, and parental country of origin, polygenic risk score for age at first birth, and first 10 principal components. ORs represent comparisons against the lowest quintile

**SFigure 4.** Odds ratios (ORs) of postpartum depression and major depression versus controls binned by PGS for cross-disorders after adjusting for maternal and paternal psychiatric history, parental country of origin, polygenic risk score for age at first birth, and first 10 principal components. ORs represent comparisons against the lowest quintile

**SFig 4a**. PGS_BD_

**SFig 4b**. PGS_SCZ_

**SFig 4c**. PGS_ASD_

**SFig 4d**. PGS_ADHD_

**STable 3.** Odds ratio of postpartum depression versus major depression by per one-standard-deviation increase in the polygenic risk scores ***among women with at least one child*** at the time of first diagnosis

| **Polygenic risk score** | **Crude OR (95% CI)** | **Adjusted OR (95% CI)** a |
| --- | --- | --- |
| **Major depression** | 1.00 (0.85–1.17) | 1.02 (0.86–1.22) |
| **Bipolar disorder** | 1.00 (0.85–1.18) | 1.04 (0.87–1.25) |
| **Schizophrenia** | 0.92 (0.78–1.08) | 0.98 (0.82–1.18) |
| **Attention deficit/ hyperactivity disorder** | 0.86 (0.73–1.00) | 0.86 (0.72–1.03) |
| **Autism spectrum disorder** | 1.05 (0.90–1.23) | 1.09 (0.92–1.28) |

^a^ adjusting for maternal and paternal psychiatric history, parental country of origin, polygenic risk score for age at first birth, age of first childbirth, and the first 10 principal components.

**STable 4.** Adjusted odds ratio of postpartum depression versus major depression by per one-standard-deviation increase in the polygenic risk scores **by the time of first postpartum depression diagnosis**

| **Polygenic risk score** | **Adjusted OR (95% CI) for early onset PPD versus MDD**  ^a^ | **Adjusted OR (95% CI)**  **for late onset PPD versus MDD** ^a^ | **P-value for difference between ORs for early onset and late onset PPD versus MDD** |
| --- | --- | --- | --- |
| **Major depression** | 1.18 (0.94–1.47) | 1.09 (0.92–1.30) | 0.584 |
| **Bipolar disorder** | 0.89 (0.71–1.11) | 0.93 (0.77–1.12) | 0.770 |
| **Schizophrenia** | 0.78 (0.62–0.99) | 0.95 (0,79–1.14) | 0.189 |
| **Attention deficit/ hyperactivity disorder** | 0.98 (0.80–1.19) | 1.18 (1.01–1.39) | 0.155 |
| **Autism spectrum disorder** | 0.93 (0.76–1.14) | 1.12 (0.97–1.30) | 0.142 |

^a^ adjusting for maternal and paternal psychiatric history, parental country of origin, polygenic risk score for age at first birth, and the first 10 principal components.

Early onset PPD: postpartum depression diagnosed within 1–90 days after delivery

Late onset PPD: postpartum depression diagnosed 91–365 days after delivery

**STable 5.** Combined adjusted odds ratio of postpartum depression versus major depression by per one-standard-deviation increase in the polygenic risk scores

| **Polygenic risk score** | **Adjusted OR (95% CI) in our study** | **Adjusted OR (95% CI) in Kiewa’s study** | **Combined OR (95% CI)** | **I-squared** |
| --- | --- | --- | --- | --- |
| **Major depression** | 1.12 (0.97–1.29) | 1.0 (0.9–1.2) | 1.06 (0.95–1.18) | 16.9% |
| **Bipolar disorder** | 0.92 (0.79–1.06) | 1.0 (0.9–1.1) | 0.97 (0.90–1.06) | 0.0% |
| **Schizophrenia** | 0.89 (0.77–1.04) | 1.0 (0.9–1.2) | 0.95 (0.84–1.06) | 17.0% |
| **Attention deficit/ hyperactivity disorder** | 1.11 (0.97–1.27) | 1.0 (0.8–1.1) | 1.06 (0.96–1.18) | 0.0% |
